# Netrin-1 improves adipose-derived stem cell proliferation, migration, and treatment effect in type 2 diabetic mice with sciatic denervation

**DOI:** 10.1186/s13287-018-1020-0

**Published:** 2018-10-25

**Authors:** Xing Zhang, Jinbao Qin, Xin Wang, Xin Guo, Junchao Liu, Xuhui Wang, Xiaoyu Wu, Xinwu Lu, Weimin Li, Xiaobing Liu

**Affiliations:** Department of Vascular Surgery, Shanghai Ninth People’s Hospital, Shanghai Jiao Tong University School of Medicine, Shanghai, 200011 China

**Keywords:** Netrin-1, Adipose-derived stem cells, Denervation, T2DM, Cell transplantation, Angiogenesis

## Abstract

**Background:**

Diabetic peripheral neurovascular diseases (DPNVs) are complex, lacking effective treatment. Autologous/allogeneic transplantation of adipose-derived stem cells (ADSCs) is a promising strategy for DPNVs. Nonetheless, the transplanted ADSCs demonstrate unsatisfying viability, migration, adhesion, and differentiation in vivo, which reduce the treatment efficiency. Netrin-1 secreted as an axon guidance molecule and served as an angiogenic factor, demonstrating its ability in enhancing cell proliferation, migration, adhesion, and neovascularization.

**Methods:**

ADSCs acquired from adipose tissue were modified by Netrin-1 gene (*NTN-1*) using the adenovirus method (N-ADSCs) and proliferation, migration, adhesion, and apoptosis examined under high-glucose condition. The sciatic denervated mice (db/db) with type 2 diabetes mellitus (T2DM) were transplanted with N-ADSCs and treatment efficiency assessed based on the laser Doppler perfusion index, immunofluorescence, and histopathological assay. Also, the molecular mechanisms underlying Netrin-1-mediated proliferation, migration, adhesion, differentiation, proangiogenic capacity, and apoptosis of ADSCs were explored.

**Results:**

N-ADSCs improved the proliferation, migration, and adhesion and inhibited the apoptosis of ADSCs in vitro in the condition of high glucose. The N-ADSCs group demonstrated an elevated laser Doppler perfusion index in the ADSCs and control groups. N-ADSCs analyzed by immunofluorescence and histopathological staining demonstrated the distribution of the cells in the injected limb muscles, indicating chronic ischemia; capillaries and endothelium were formed by differentiation of N-ADSCs. The N-ADSCs group showed a significantly high density of the microvessels than the ADSCs group. The upregulation of AKT/PI3K/eNOS/P-38/NF-κB signaling pathways and secretion of multiple growth factors might explain the positive effects of Netrin-1 on ADSCs.

**Conclusion:**

The overexpression of Netrin-1 in ADSCs improves proliferation, migration, and treatment effect in type 2 diabetic mice with sciatic denervation, which directs the clinical treatment of patients with DPNVs.

## Background

Diabetes mellitus (DM) is a common and frequently occurring disease worldwide [1, 2]. As the most common chronic complication in diabetic patients, diabetic peripheral neurovascular disease (DPNV) is characterized by high incidence, early onset, long duration, and refractoriness [3]. Some DPNV patients develop diabetic foot as well as critical limb ischemia (CLI) and face severe consequences of amputation and even life-threatening situations [4]. Although conservative in nature, treatments, such as revascularization, percutaneous transluminal angioplasty, and amputation, can improve the prognosis of patients slightly; however, the long-term curative effect is not satisfactory, and hence, their clinical application of these treatments is limited [5, 6]. The combination of angiopathy and neuropathy is the main reason of refractory DPNV; therefore, a positive and effective method for treating the disease is an urgent requisite.

With the progress in regenerative medicine and studies in stem cells, the technique of stem cell transplantation increases the formation of collateral blood vessels in ischemic limbs and restores the blood flow [7, 8]. With an abundant natural source in T2DM patients due to their obesity, adipose-derived stem cells (ADSCs) have the advantage of convenience and highly expressed stemness, rendering them as the optimal therapy for ischemic disease than endothelial progenitor cells (EPCs) and bone marrow-derived stem cells (BMSCs). Nevertheless, the treatment efficiency of ADSCs was impaired by T2DM, primarily reflected in the ability of proliferation, adhesion, and angiogenic potential [9–12]. Hence, the mechanisms underlying the survival, differentiation of ADSCs under hyperglycemia, and promotion of angiogenesis after DPNV injury are yet to be elucidated.

Previous studies found that DPNV is a mixed lesion with vascular, nerve, and tissue damages [13]. Also, the nerves and blood vessels exhibited similar complex branching and growth patterns. In addition, these nerves and blood vessels follow the same migration path and the route to the same target organ or site. The similarities between angiogenesis and axonal growth demonstrate that they may be regulated by some common signal molecules [14]. Jones and Li showed that some signaling molecules can regulate the process of nerve and blood vessel development [15]. Netrin-1 is the first identified axon-guiding factor. Netrin-1 and G-netrin share homology with laminin γ chain and contain a laminin-VI region, three EGF-like regions of integrin-V, and a heparin-bound carboxyterminal (C region) [16]. Another study found that Netrin-1 participates in the functional activity of not only the nerves but also the blood vessel systems. Ding et al. demonstrated that Netrin-1 not only promotes neuronal migration and secretion in the central nervous system [17, 18] but also regulates the survival, adhesion, migration, proliferation, and differentiation of the endothelial cells and stem cells in non-nerve tissue and inhibits their apoptosis [19]. Wilson et al. systematically studied zebrafish and mammals and found that Netrins stimulate angiogenesis [20]. Netrin-1 activates Src/FAK/paxillin-related signaling pathway via binding to the UNC5H receptor to promote vascular endothelial cell adhesion, migration, and proliferation for the formation of a new capillary network that could be hindered by inhibiting the zebrafish *NTN-1* mRNA. Lu et al. also found that Netrins can stimulate angiogenesis in mammals and accelerate that of the ischemic tissue. This process relies on Netrin-1 receptor DCC to regulate the ERK/eNOS signaling pathway [21]. Brunet et al. demonstrated that Netrin-1, Netrin-4, and vascular endothelial growth factor (VEGF) promoted angiogenesis; however, Netrin-1 is superior in promoting the dual role of endothelial cell differentiation and nerve injury recovery [22]. Furthermore, the neurotrophic factor Netrin-1 is involved in nerve growth and angiogenesis; it also enhances mitosis, migration, and adhesion of endothelial cells at different stages of the human blood vessel and microvasculature. Hence, we supposed that the viability, migration, and multi-differentiation of ADSCs under hyperglycemia condition could be improved by the overexpression of Netrin-1 via gene transfection.

In the current study, we transfected the ADSCs with the *NTN-1* gene (N-ADSCs) and examined their proliferation, migration, adhesion, and apoptosis under high-glucose condition. Subsequently, the N-ADSCs were transplanted into sciatic denervated mice (db/db) with T2DM. The laser Doppler perfusion index, immunofluorescence, and histopathological assay were used to analyze the treatment efficiency. In addition, Netrin-1-mediated mechanism of ADSCs underlying the enhancement of proliferation, migration, adhesion, and differentiation was also elucidated.

## Methods

### Animals

Wild-type (WT) C57/BL mice and type 2 diabetic mice (BKS. Cg-m +/+Lepr^db^) were purchased from Shanghai Research Center for Model Organisms (Shanghai, China). All animal experiments were approved by the Animal Ethics Committee of Shanghai Ninth People’s Hospital, Shanghai Jiao Tong University School of Medicine. The blood glucose level of the diabetic and hyperglycemic mice was characterized as ≥ 16.67 mmol/L, and only these mice were used for the subsequent in vivo studies.

### Isolation, culture, and characterization of ADSCs

ADSCs were obtained from the subcutaneous adipose tissues of the inguinal area of 4-week-old WT C57/BL6 mice and maintained in low glucose (5 mmol/L) Dulbecco’s modified Eagle’s medium (DMEM), supplemented with 10% fetal bovine serum (FBS), 100 U/mL penicillin, and 100 mg/mL streptomycin at 37 °C in a 5% CO_2_ incubator [23]. DMEM with high glucose (33.3 mmol/L) was utilized to culture ADSCs in order to mimic the in vivo hyperglycemia condition in T2DM for the subsequent experiments. The following experiments adopted ADSCs between passages 3 and 5. The phenotype of the ADSCs was determined by flow cytometry. Briefly, passage 3 ADSCs were obtained and washed using phosphate-buffered solution (PBS), after incubation with phycoerythrin-conjugated anti-mouse antibodies against CD90, and fluorescein isothiocyanate-conjugated anti-mouse antibodies against Sca-1 for 30 mins at 4 °C in the dark. The negative control group utilized the isotype antibodies. The cells were washed three times and harvested for flow cytometry (Beckman Coulter, Fullerton, CA, USA).

### Gene transfection of ADSCs

Adenovirus was adopted for a stable transfection in order to establish the Netrin-1 recombinant adenovirus construct. Firstly, RT-PCR was used to clone the *NTN-1* gene; pDC316-CMV carrying the green fluorescent protein (*GFP*) reporter gene was used as a shuttle vector. Secondly, after nuclease digestion, sequencing analysis identified pDC316-Netrin-1. Next, Lipofectamine 2000 was used for transfecting the adenovirus-packaging plasmid pBHGlox_E1.3Cre into the human embryonic kidney cell line 293 (HEK293). Consequently, GFP-Netrin-1, a recombinant adenovirus vector carrying Netrin-1, was formed in HEK293 cells and confirmed by PCR. After purification, amplification, and titration, GFP-Netrin-1 was transfected into mice ADSCs, and Western blotting and real-time PCR were employed to verify the expression of Netrin-1. Mice ADSCs, infected with GFP, served as the control group. Furthermore, multiplicity of infection (MOI) was set under 250, 500, and 1000, and the duration was set to 24 h and 48 h. The optimal MOI and duration for transfecting ADSCs were explored for maximal transfection efficiency.

### Cell viability assay

The proliferation of ADSCs and N-ADSCs was assessed using Cell Counting Kit-8 (CCK-8; Dojindo Laboratories, Kumamoto, Japan, http://www.dojindo.cn) following the manufacturer’s instructions. The cells were harvested and dispensed in a 96-well plate containing high-glucose DMEM at 2–3 × 10^3^ cells/well. At 1, 2, 3, and 4–7 days, we added 10 μL/well CCK-8 reagent and incubated the cells at 37 °C for 2 h, before measuring the absorbance at 450 nm using a microplate spectrophotometer (Varioskan; Thermo Fisher).

### Effect of Netrin-1 on ADSCs apoptosis under high glucose

ADSCs and N-ADSCs were incubated in high-glucose condition for 48 h. The rate of apoptosis was analyzed with the Annexin V apoptosis detection kit (BD Bioscience, New York, USA) as described previously [11]. After incubation, we harvested the floating and adherent cells by trypsinization and used Annexin V (Thermo Fisher) and propidium iodide (PI) to stain the cells for 15 min, followed by flow cytometry (Beckman Coulter) to quantify the percentage of apoptotic cells (Annexin V^+^/PI^−^). Finally, N-ADSC and ADSC lysates were subjected to Western blotting with antibodies against Bcl-2, Bax, and β-actin (1:500; Abcam, Cambridge, UK).

### Wound healing assay, cell migration, adhesion, and tube formation assays under high-glucose condition

Scratch wound healing assays were conducted by scratching the cell layer with 1 mL pipette tips, followed by washing the wells two times with PBS. Subsequently, the cells were cultured in high-glucose DMEM for 24 h, and the images recorded with an Olympus inverted microscope (Olympus Microscopes, Tokyo, Japan, http://www.olympus-ims.com) at 0, 12, and 24 h. Photoshop software (Adobe Systems Inc., San Jose, CA, USA, http://www.adobe.com) was utilized to analyze the wound gap area; six visual fields were selected randomly to obtain an average. The data were normalized to the mean of the control.

Migration assays were conducted using a Boyden chamber and an 8-μm polyethylene terephthalate (PET) membrane (R&D Systems Inc., Minneapolis, MN, USA). 2 × 10^3^ ADSCs were suspended in 100 μL high-glucose DMEM supplemented with 0.5% FBS and added into the upper chamber, while 600 μL medium was added into the lower chamber. After incubation for 24 h at 37 °C and 5% CO_2_, the filter was removed, and cells in the upper chamber were removed using a cotton tip. Four percent paraformaldehyde was used to fix the ADSCs that migrated into the lower chamber, and 1% crystal violet in 2% ethanol was used for staining the cells. The experiments were repeated three times.

Adhesion assays were conducted by incubating ADSCs and N-ADSCs in a 6-well plate at a density of 1 × 10^5^ cells/well for 30 min at 37 °C in high-glucose DMEM. After three washes, the cell nuclei were stained with DAPI (Dako) and the number of cells counted using a hemocytometer under immunofluorescence microscope. Each experiment was conducted in three wells and repeated in triplicate.

Tube formation assays were conducted by culturing ADSCs and N-ADSCs in high-glucose DMEM containing 1% FBS, 100 U/mL penicillin G, and 100 mg/mL streptomycin for 48 h and plating on Matrigel (BD Biosciences, San Jose, CA, http://www.bdbiosciences.com) in 24-well plates. After incubating for 12 h at 37 °C in 5% CO_2_, the images were acquired with a microscope, and the cumulative tubular growth analyzed using ImageJ Pro Plus software (NIH, Bethesda, MD, http://www.imagej.nih.gov).

### Induction of hind limb sciatic denervation and cell transplantation

All animal experiments were approved by the Animal Ethics Committee of Shanghai Ninth People’s Hospital, Shanghai Jiao Tong University School of Medicine. Male type 2 diabetic mice (BKS. Cg-m +/+Lepr^db^) (18–20 weeks old, *n* = 18; Shanghai Research Center for Model Organism, China) were divided into three groups; subsequently, the animals were anesthetized using pentobarbital sodium (0.5 mg/g). For each mouse, the left hindquarter was shaved intensively. The sciatic nerve was separated under direct vision, and a piece of nerve 1.0 cm in length was removed as described previously [24, 25]. The muscle was sutured with 5-0 nylon sutures and the skin with interrupted 3-0 nylon sutures. After 24 h, three different sites on the denervated hindlimb (gastrocnemius, gracilis, and quadriceps muscles) were injected with 100 μL serum-free medium containing 10^5^ cells. The mice were randomly divided into three groups: the ADSCs group (*n* = 6), the N-ADSCs group (*n* = 6), and the control group (*n* = 6) that received PBS. A laser Doppler perfusion imager (moorFLPI; Moor Instruments, Devon, UK) was adopted to assess the blood flow non-invasively on days 7, 14, and 28 after transplantation.

### Immunofluorescence and immunohistochemistry staining

In order to examine the survival and differentiation of N-ADSCs in the denervated muscles, we anesthetized the mice and performed the perfusion fixation on day 28. Then, the muscles were removed and histological and immunohistochemical assays performed. The frozen slices were subjected to immunofluorescent and immunohistochemistry staining. The muscle slices were stained with CD31 (PECAM-1; Abcam) and the nuclei with DAPI (Dako) as described previously [7].

### Evaluation of signaling pathway by Western blotting

Total protein was isolated from N-ADSCs and ADSCs cultured under high glucose for 48 h in vitro, and the muscle tissues of db/db mice denervated limbs injected with ADSCs and N-ADSCs, respectively. The total protein in tissue samples or cell lysates was quantified, electrophoresed, and transferred to PVDF membranes that were probed with appropriate antibodies at 4 °C overnight as follows: anti-Akt and P-AKT antibody, anti-PI3K and P-PI3K antibody, anti-P38 and P-P38 antibody, anti-eNOS and P-eNOS antibody, anti-NF-κB and P-NF-κB antibody, anti-JNK antibody, anti-ERK1/2 antibody, and anti-β-actin antibody (1:500; Abcam). The Odyssey infrared imaging system (LI-COR, Lincoln, NE, USA) was used to quantify the relative integral density of the immunoreactive bands.

### Estimation of paracrine factors by enzyme-linked immunosorbent assay

The levels of vascular endothelial growth factor (VEGF), basic fibroblast growth factor (b-FGF), hepatocyte growth factor (HGF), tumor necrosis factor-α (TNF-α), platelet-derived growth factor (PDGF), epidermal growth factor (EGF), insulin-like growth factor (IGF-1), and Netrin-1 released from ADSCs and N-ADSCs into the high-glucose DMEM medium at 48 h were examined by ELISA kit, following the manufacturer’s instructions (R&D Systems Inc.). The high-glucose DMEM medium was utilized as a control. Every experiment was conducted more than three times in three wells.

### Statistical analysis

Data are demonstrated as the mean values ± standard deviation. Student’s *t* test and one-way analysis of variance were used to compare and analyze the quantitative values; statistical significance is defined as *P < 0.05. Each experiment was conducted more than three times.

## Results

### Characterization and flow cytometry analysis of ADSCs

**Fig. 1:**
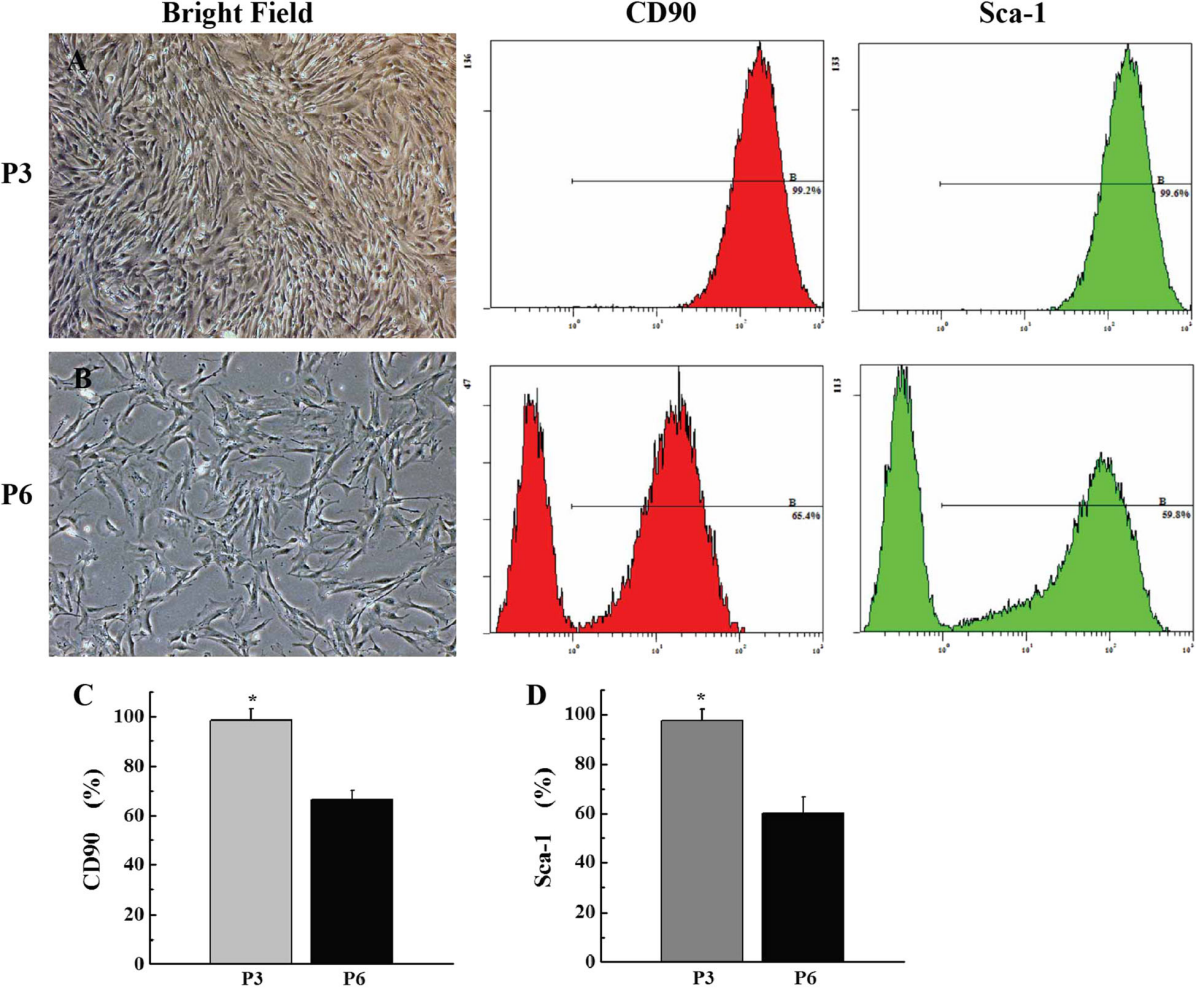
Characterization and flow cytometry analysis of ADSCs. **a** P3 ADSCs adopted a uniform fibroblast-like, spindle-shaped population. **b** P6 ADSCs adopted increased aging or dedifferentiated shape with irregularity. Flow cytometry revealed that the level of CD90 and Sca-1 surface antigens of P3 and P6 ADSCs were reduced gradually with statistically significant differences (*P < 0.05; **c**, **d**) Scale bar = 100 μm. P3, passage 3; P6, passage 6; ADSC, adipose-derived stem cell

ADSCs were isolated from inguinal adipose tissues and expanded quickly. P3 ADSCs showed a pattern of fibroblast-like, spindle-shaped population (Fig. 1a). Phenotypic analysis by flow cytometry demonstrated that the P3 ADSCs were strongly double-positive for the stem cell surface antigens, such as CD90 (99.2 ± 3.73%) and Sca-1 (99.6 ± 3.08%) (Fig. 1), and negative for CD11b, CD31, CD34, CD45, CD133, and MHC-II (data not shown). However, P6 ADSCs adopted aging or dedifferentiation with irregularity by increasing the culture time and passages in vitro (Fig. 1b). Flow cytometry analysis revealed that the level of stem cell-specific marker CD90 (65.4 ± 3.42%) and Sca-1 (59.8 ± 3.15%) surface antigen of ADSCs (Fig. 1c, d) was reduced gradually with a statistically significant difference (*P* < 0.05).

### *NTN-1* gene transfection into ADSCs

**Fig. 2:**
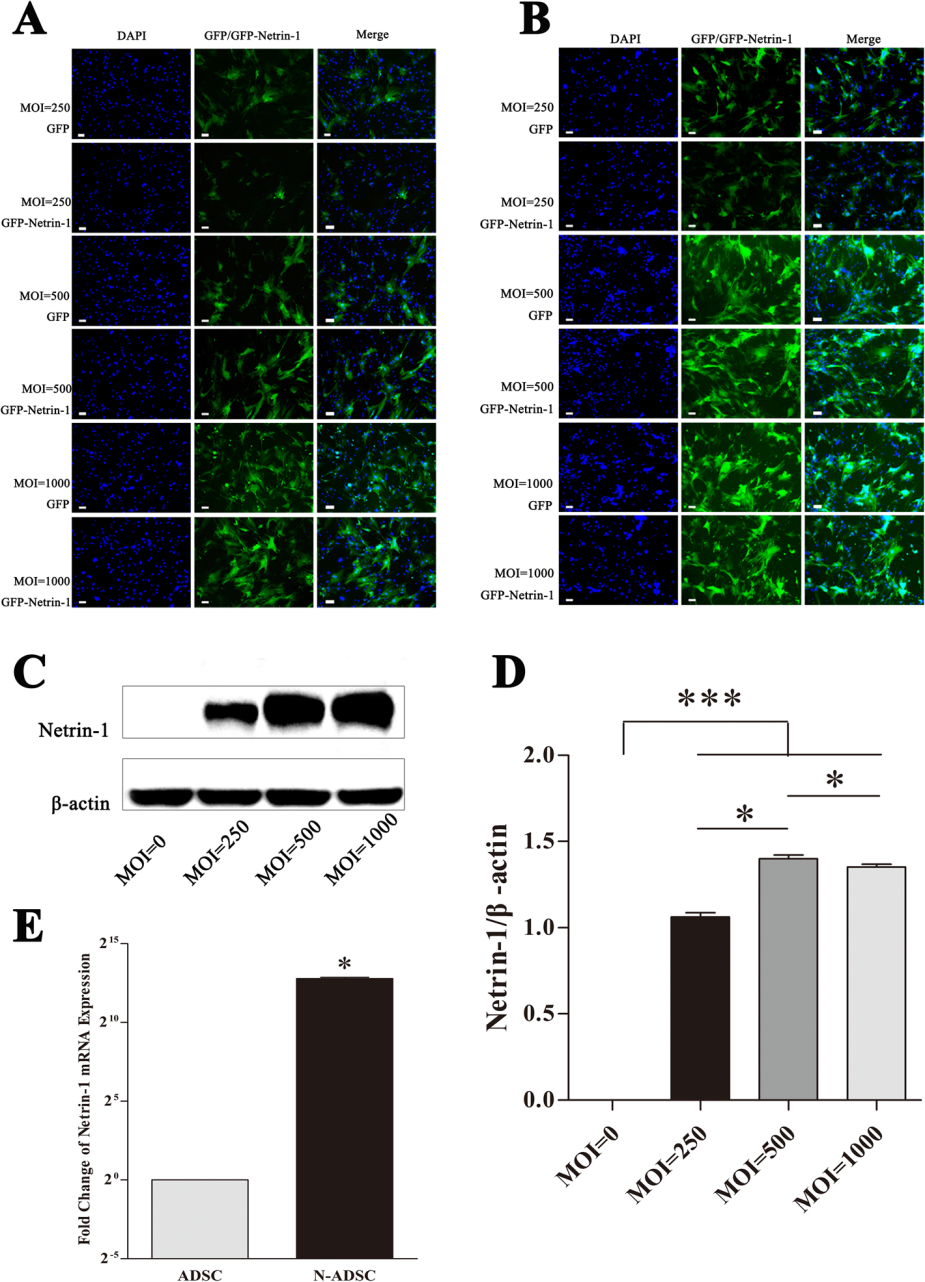
Gene transfection was adopted to overexpress *NTN-1* by ADSCs. **a**, **b** Slight difference was observed in the transduction ratio between *NTN-1* and *GFP* in ADSC cells. **c**–**e** Western blot analysis, statistical analysis, and PCR analysis verified a significantly high expression of Netrin-1 in N-ADSCs group as compared to no expression in the ADSCs group (*P* < 0.05). Scale bar = 100 μm. *P < 0.05. ADSC, adipose-derived stem cell; GFP, green fluorescent protein; N-ADSC, Netrin-1-transfected ADSC

In comparison to other viral vector systems, adenovirus vectors have a wide host range and are low-pathogenic in humans. These vectors can infect and express the target gene in proliferating and non-proliferating cells, not integrate into the chromosome, do not have mutagenicity, and can express multiple genes simultaneously; in addition, they can be produced in high titers and can make the transgene express for a prolonged duration with little side effects [26]. *NTN-1* was transduced into ADSCs by adenovirus; the best MOI was 500, and the duration for transfecting ADSCs was 48 h to achieve maximum transfection efficiency (Fig. 2). The transduction ratio did not vary markedly between Netrin-1 and GFP in ADSC cells (Fig. 2a, b). Western blot, statistical analysis, and PCR demonstrated a significantly high expression of Netrin-1 in the N-ADSCs group and almost no expression in the ADSCs group (*P* < 0.05) (Fig. 2c–e). Thus, a si-RNA-*NTN-1* group was not required as blank control. Herein, we successfully established a system, wherein the *NTN-1* gene was transfected with high efficiency and expressed in ADSCs.

### CCK-8 assay of the viability of N-ADSCs under high glucose

**Fig. 3:**
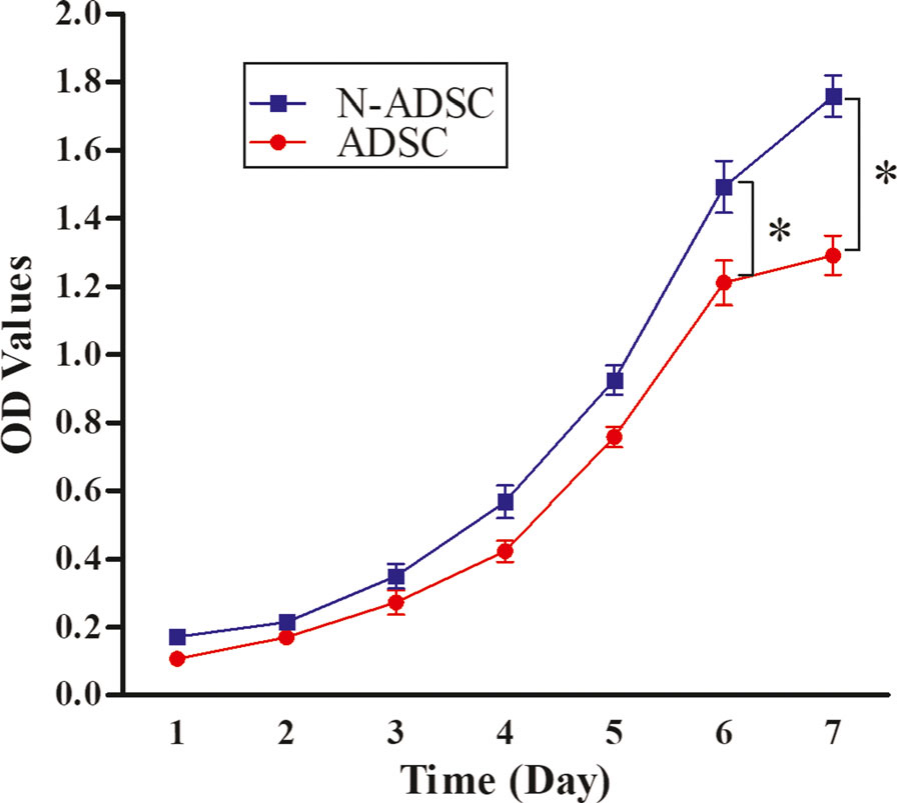
Cell viability of N-ADSCs. Cells transfected with Netrin-1 exhibited a higher proliferation rate than the untreated negative control ADSCs from days 3–7 (*P* < 0.05, *n* = 5); *P < 0.05. ADSC, adipose-derived stem cell; OD, optical density; N-ADSC, Netrin-1-transfected ADSC

From days 1 to 7, the proliferation of ADSCs and N-ADSCs was assessed using a CCK-8 assay to explore the effect of Netrin-1 on cell viability (Fig. 3). Furthermore, within the first 3 days, ADSCs and N-ADSCs were demonstrated to be relatively stationary in growth. From days 3 to 7, both cells progressed into a logarithmic growth pattern. In comparison to ADSCs, N-ADSCs demonstrated a significantly higher growth rate from days 3 to 7 (*P* < 0.05, *n* = 5). The present study confirmed the promoting effect of Netrin-1 on ADSC proliferation.

### Effects of Netrin-1 on ADSC apoptosis under high glucose

**Fig. 4:**
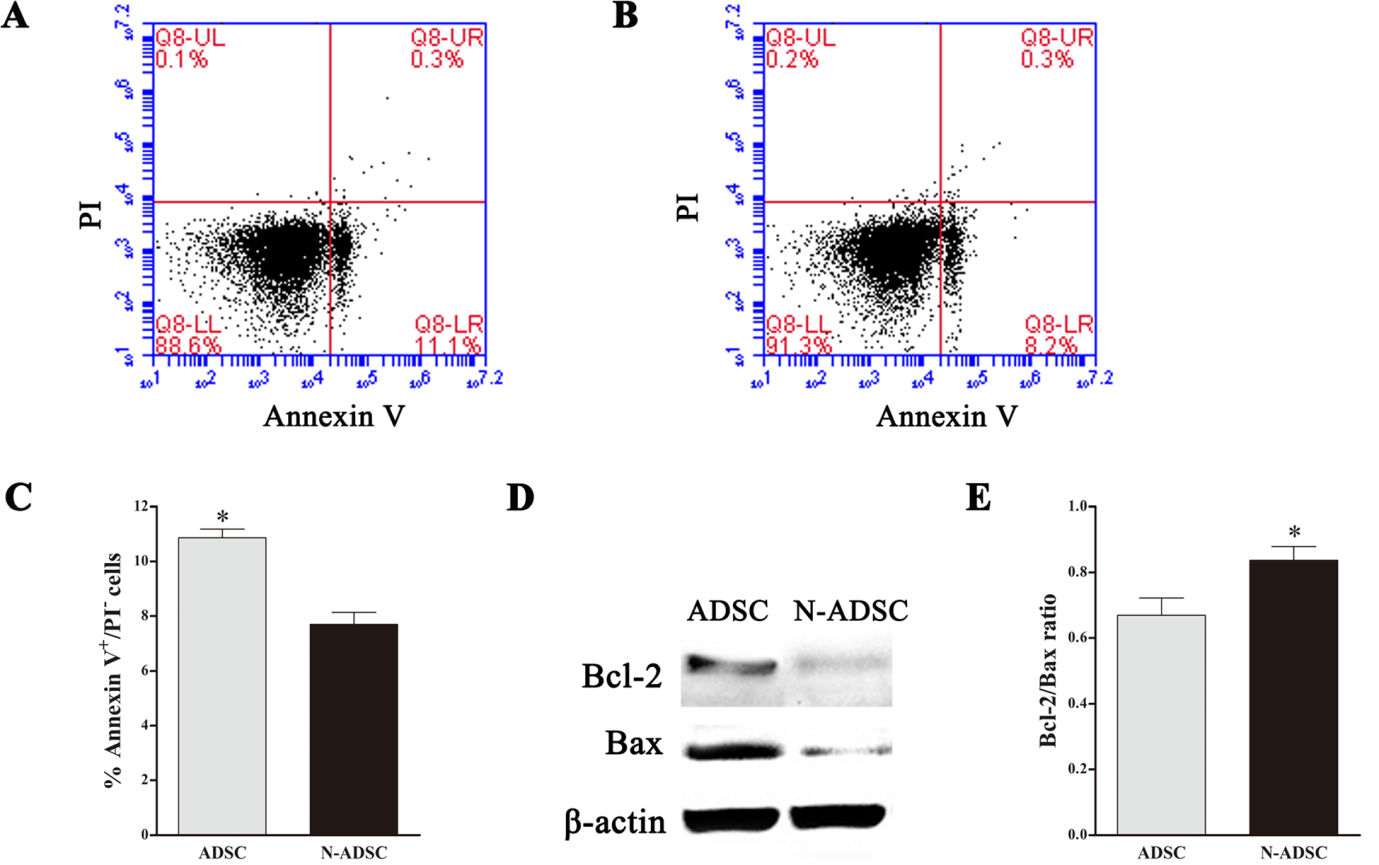
Effects of Netrin-1 on the apoptosis of ADSCs under high glucose. **a**, **b** Apoptosis of GFP-ADSCs transfected with or without Netrin-1 under high glucose was analyzed by flow cytometry. **c** The rate of apoptosis indicated that overexpression of Netrin-1 significantly improved the viability of ADSCs. **d**, **e** Western blotting and statistical analysis revealed that the ratio of Bcl-2/Bax was significantly higher in N-ADSCs than ADSCs under high-glucose condition; *P < 0.05. ADSC, adipose-derived stem cell; GFP green fluorescent protein; N-ADSC, Netrin-1-transfected ADSC

In this study, we used a high-glucose condition to mimic the hyperglycemia of type 2 diabetic patients. Previous studies discovered that hyperglycemia had a negative effect on the proliferation and apoptosis of stem cells, ADSCs, gained from type 2 diabetic patients, and mice demonstrated the low viability and high apoptosis rate [9–12]. In order to explore the effect of Netrin-1 on ADSC apoptosis under high glucose, we stained the cells with Annexin V/PI and analyzed by flow cytometry. The apoptotic percentage was 7.7 ± 0.44% in the N-ADSCs group as compared to 10.9 ± 0.32% (Fig. 4a, b) in the ADSCs group, which was significantly different (*P* < 0.05) (Fig. 4c). Western blotting and statistical analysis demonstrated that the Bcl-2/Bax ratio of N-ADSCs was significantly higher than that of ADSCs under high glucose (Fig. 4d, e), thereby indicating that the apoptosis rate was inhibited in N-ADSCs as compared to ADSCs under high glucose in vitro. Taken together, the transfection of Netrin-1 into ADSCs promoted viability and anti-apoptosis.

### Effects of Netrin-1 on migration, adhesion, and tube formation of ADSCs under high glucose

**Fig. 5:**
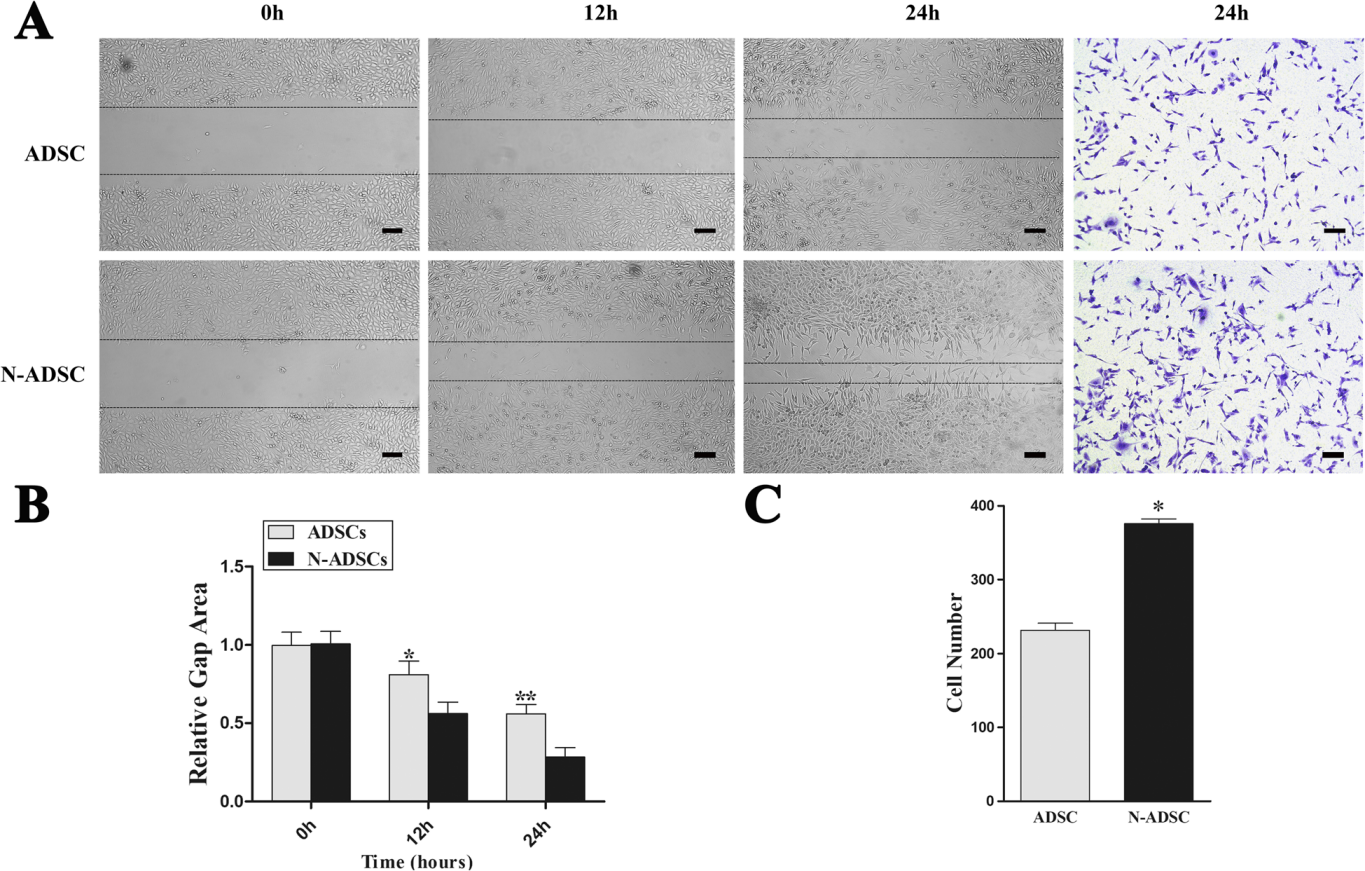
Effects of Netrin-1 on the migration of ADSCs. **a** Representative images of wound healing and Transwell assays after 24 h culture under high-glucose conditions. **b** Wound healing assay showed that the relative gap area was significantly smaller in the N-ADSCs group than in the ADSCs group under high-glucose conditions. **c** Transwell assay showed that a significant number of cells had migrated in the N-ADSCs group than in the ADSCs group under high-glucose conditions. *P < 0.05, Scale bar = 100 mm. ADSC, adipose-derived stem cell; N-ADSC, Netrin-1-transfected ADSC

As the injected stem cells in vivo would gradually migrate to the injured area, we examined the effect of Netrin-1 overexpression on the migration ability of ADSCs under high glucose. During the wound healing assay, N-ADSCs healed significantly faster than ADSCs at 12 and 24 h (relative gap area, *P* < 0.05) (Fig. 5a, b). The outcome of the Transwell assay (Corning Inc.) demonstrated that the N-ADSCs migrated significantly more than the ADSCs through the membrane (ADSCs, 231.67 ± 9.50 cells/field; N-ADSCs, 375.67 ± 6.51 cells/field; *P* < 0.05) (Fig. 5a, c). In summary, our results verified the overexpression of Netrin-1 promoted the migration ability of ADSCs under high-glucose condition.

**Fig. 6:**
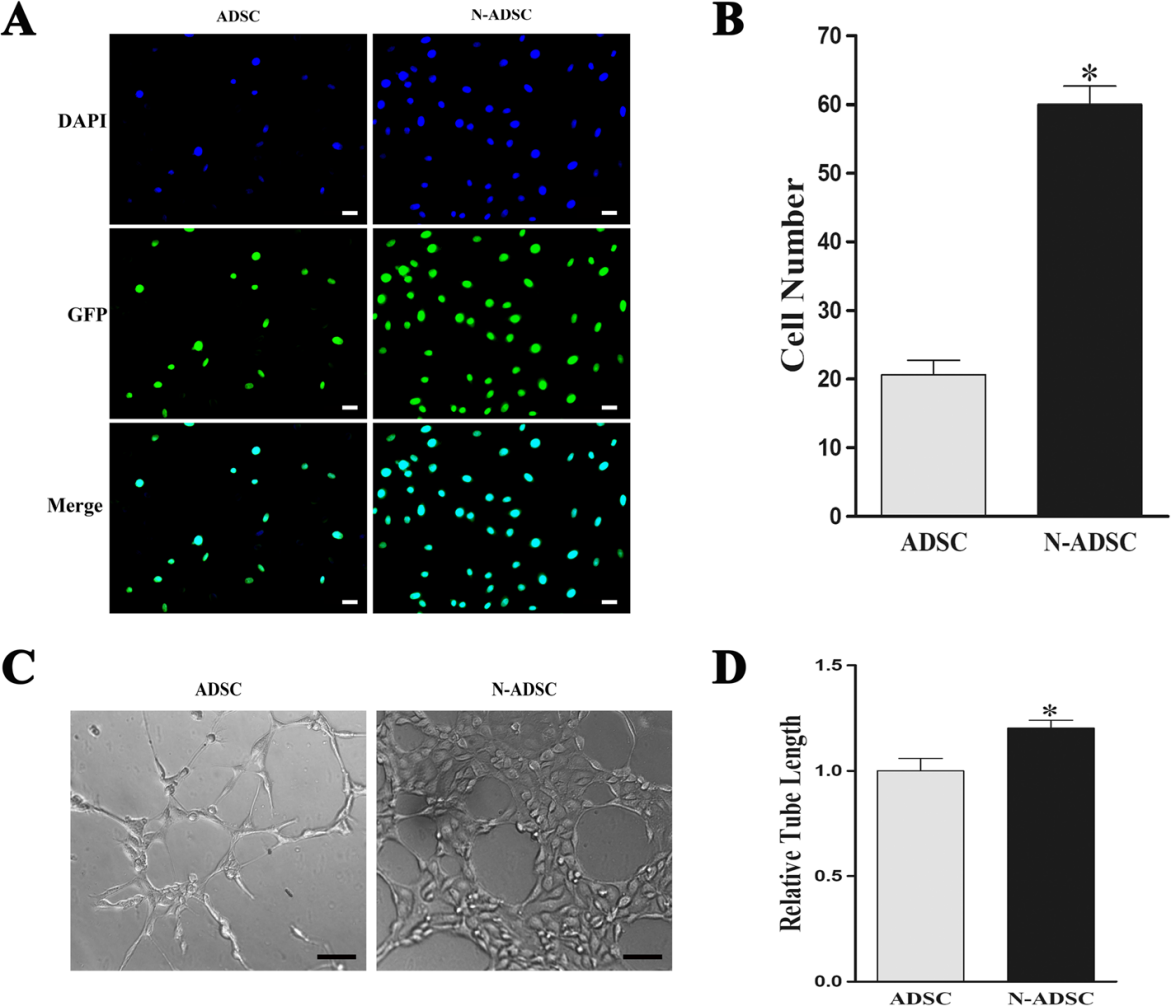
Effects of Netrin-1 on adhesion and the proangiogenic ability of ADSCs under high glucose. **a** A large number of attached cells was observed in the N-ADSC group as compared to that in the ADSCs control group. **b** Quantitative assays indicated that Netrin-1 overexpression effectively enhanced the adhesion of ADSCs. **c** Representative images of tube formation of N-ADSCs and ADSCs seeded onto Matrigel under high glucose for 12 h. **d** Cumulative tubular length of N-ADSCs was higher than that of ADSCs; *P < 0.05, Scale bars = 100 mm. ADSC, adipose-derived stem cell; N-ADSC, Netrin-1-transfected ADSC

For the adhesion test, the cells attached to the wells were round in shape and were observed as blue and green fluorescence under a fluorescence microscope (Fig. 6). Statistical analysis showed that the N-ADSCs group had a higher adhesion cell number than that of the ADSCs group (ADSCs, 20.67 ± 2.08 per field; N-ADSCs, 60.00 ± 2.65 per field, *P* < 0.05) (Fig. 6a, b). The current results showed that the overexpression of Netrin-1 significantly elevated the adhesion ability of ADSCs.

Tube formation assay demonstrated abundant capillary formation in N-ADSCs than that in ADSCs, while the cumulative tubular length of N-ADSCs was higher than that of ADSCs in high-glucose culture medium (1.21-fold, *P* < 0.05) (Fig. 6c, d). The current results demonstrated that the proangiogenic ability was elevated by the overexpression of Netrin-1 in ADSCs.

### Effects of N-ADSCs on blood perfusion and histological staining of type 2 diabetic mice of sciatic denervation

**Fig. 7:**
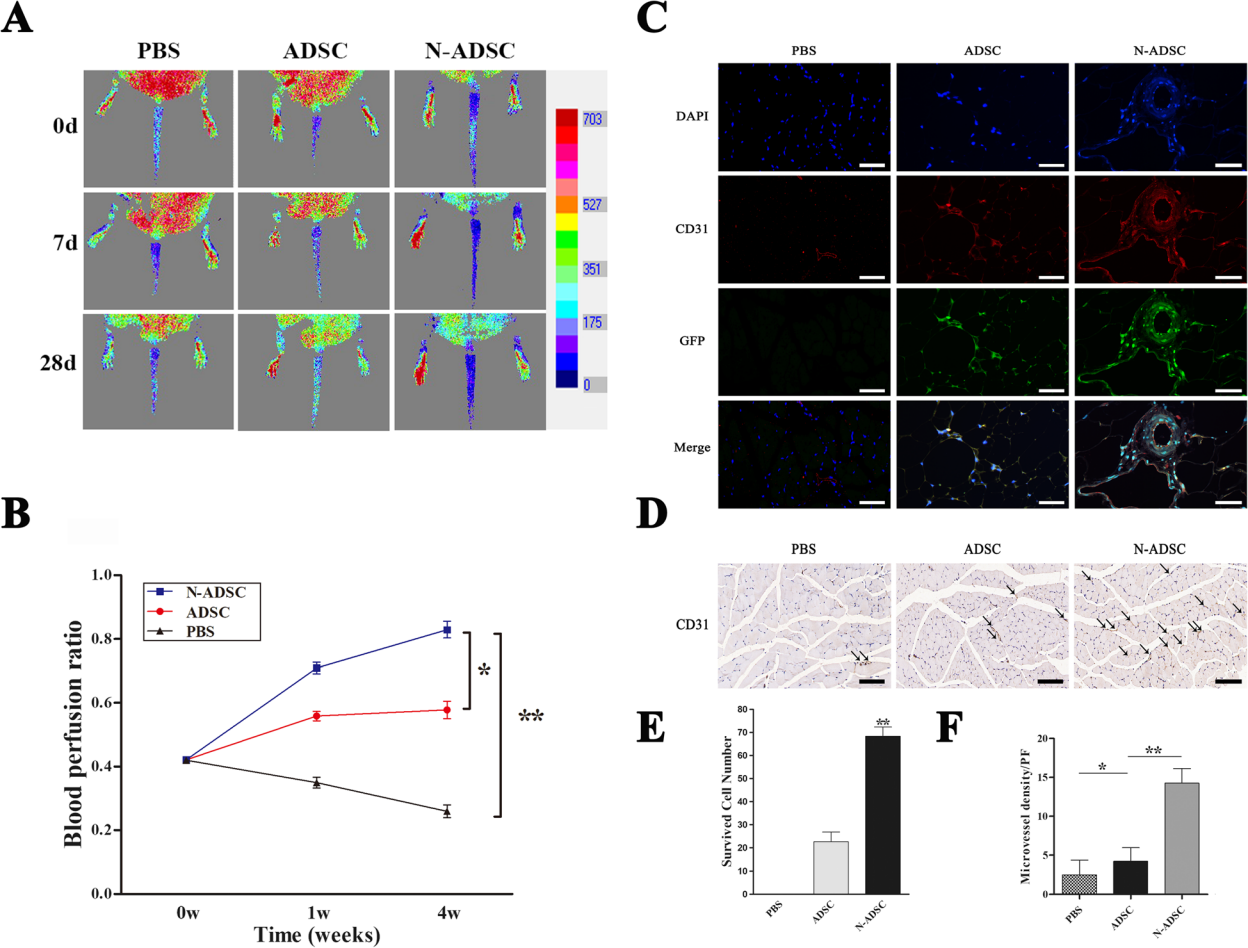
Effects of transplantation of N-ADSCs on blood flow and histological staining. **a** Representative color laser Doppler images of the superficial blood flow in the denervated hindlimbs were acquired on days 0, 7, and 28 post-transplantation. **b** The blood perfusion index was significantly higher in the N-ADSCs group as compared to that in the ADSCs and PBS control groups over 4 weeks. **c** Representative fluorescent staining for CD31 (red), ADSCs (green), and nuclei with DAPI (blue) in the PBS, ADSC, and N-ADSC groups. **d** Representative immunohistochemical staining for CD31 in the PBS, ADSC, and N-ADSCs groups. **e** Statistical analysis indicated that the survived ADSCs were significantly higher in N-ADSCs than in ADSCs (*P* < 0.05). **f** Quantitative analysis revealed that microvessel densities were significantly higher in the N-ADSCs than in the ADSCs and PBS control groups (*P* < 0.01). Scale bar = 100 μm; *P < 0.05, **P < 0.01. ADSC, adipose-derived stem cell; PBS, phosphate-buffered solution; N-ADSC, Netrin-1-transfected ADSC

Previous studies revealed that sciatic denervation resulted in the vascular bed remodeling and progressive loss of capillaries and impaired the arteriogenesis and ischemic recovery, which accelerated the deterioration of DPVN in type 2 diabetic mice [24, 25]. Herein, we observed the effect of Netrin-1 on ADSCs in type 2 diabetic mice of sciatic denervation, which showed a pattern of chronic lower limb ischemia. After cell transplantation, the condition of the hindlimb blood flow was imaged by color laser Doppler on days 0, 7, 14, and 28 (Fig. 7a). Both the N-ADSCs and ADSCs groups showed a significantly higher laser Doppler perfusion index than the PBS control group. Moreover, the N-ADSCs group demonstrated a significantly higher laser Doppler perfusion index than the ADSCs group within 4 weeks (ADSCs group, 0.58 ± 0.03; N-ADSCs group, 0.83 ± 0.03; PBS group, 0.26 ± 0.02; *P* < 0.05) (Fig. 7b). After 28 days post-injection of cells, no tumor or teratoma was formed in the denervated ischemic muscles or other organs. Cells expressing GFP were assessed in the injected limb muscles by immunofluorescence microscopy. CD31 (red), DAPI (blue) double staining, and the already transfected GFP (green) verified the migration of cells towards chronic ischemic sites and impaired vascular areas, some of which eventually differentiated into vascular endothelial cells (Fig. 7c). The result of the present study demonstrated the participation of N-ADSCs in the recovery of injured vascular structure and revascularization. Statistical analysis indicated a significantly higher number of survived cells in the N-ADSCs group than in the ADSCs and PBS control groups (ADSCs group, 22.67 ± 4.16 per field; N-ADSCs group, 68.33 ± 4.04 per field, *P* < 0.01) (Fig. 7e). Moreover, immunohistochemistry analysis revealed that N-ADSCs significantly increased the microvessel densities in the denervated hindlimbs as compared to ADSCs and PBS groups (PBS group, 2.50 ± 1.91 per field; ADSCs group, 4.25 ± 1.71 per field; N-ADSCs group, 14.25 ± 1.89 per field, *P* < 0.05) (Fig. 7d, f). The current results demonstrated the crucial role of Netrin-1 in the in vivo survival, migration, and differentiation of ADSCs.

### Effect of Netrin-1 on the secretion of cytokines and growth factors in ADSCs

**Fig. 8:**
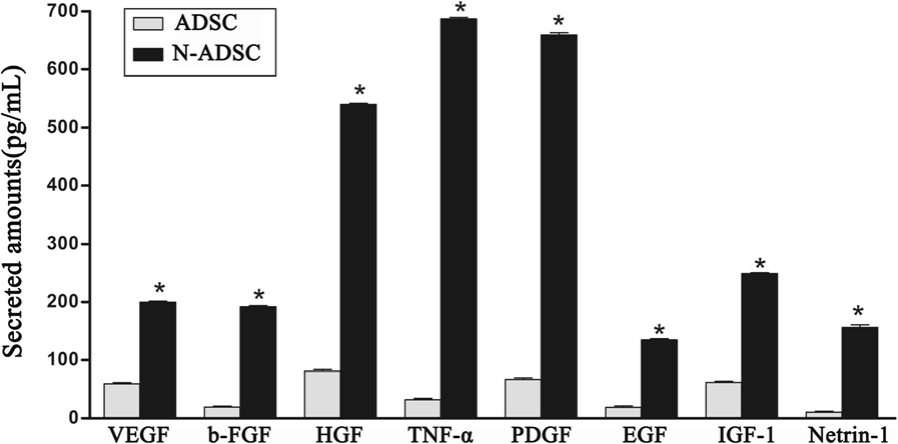
Cytokines and growth factors in N-ADSCs. ELISA showed that the levels of cytokines and growth factors, including VEGF, b-FGF, HGF, TNF-α, PDGF, EGF, IGF-1, and Netrin-1, were upregulated in N-ADSCs as compared to ADSCs; *P < 0.05. ADSC, adipose-derived stem cell; N-ADSC netrin-1-transfected ADSC; VEGF vascular endothelial growth factor; b-FGF, basic fibroblast growth factor; HGF, hepatocyte growth factor; TNF-α, tumor necrosis factor-alpha; PDGF, platelet-derived growth factor; EGF, epidermal growth factor; IGF-1, insulin-like growth factor-1

ADSCs and N-ADSCs were cultured in high-glucose media for 48 h, following which the supernatants were collected and analyzed by ELISA. The expression of cytokines and growth factors, such as VEGF, b-FGF, HGF, TNF-α, PDGF, EGF, IGF-1, and Netrin-1, was higher in the N-ADSCs group than in the ADSCs group (Fig. 8). Furthermore, Netrin-1 upregulated the paracrine of ADSCs to enhance revascularization in vivo.

### Signaling pathways in N-ADSCs

**Fig. 9:**
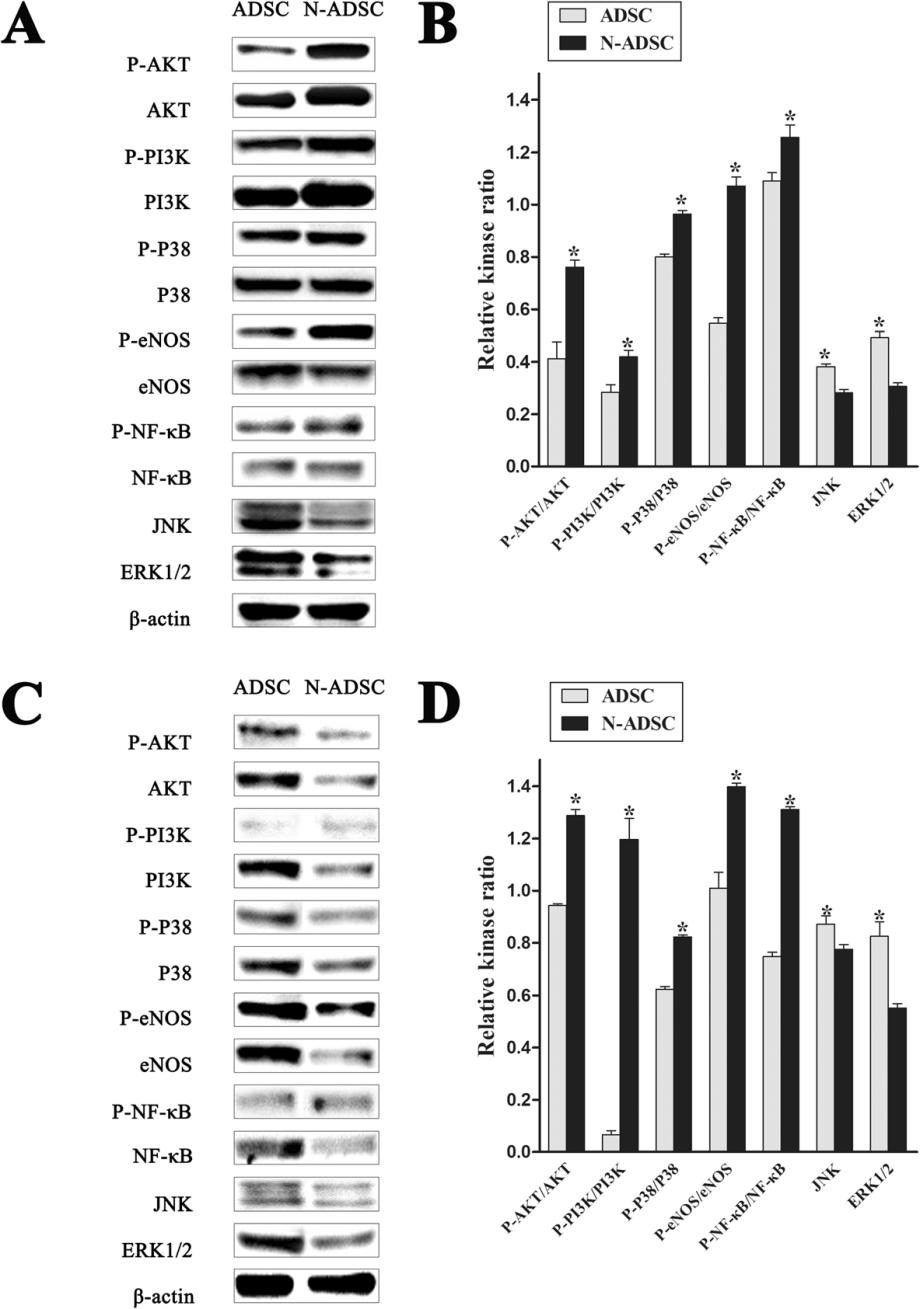
Signaling pathway of Netrin-1 on ADSCs in vitro and in vivo. **a** Western blotting of P-AKT, AKT, P-PI3K, PI3K, P-P38, P38, P-eNOS, eNOS, P-NF-κB, NF-κB, JNK, and ERK1/2 in vitro. **b** Statistical analysis demonstrated that the phosphorylation of the signaling pathway of PI3K/AKT/eNOS/P-38/NF-κB was highly upregulated in the N-ADSCs group as compared to the ADSCs group in vitro, but not JNK or ERK1/2. **c** Western blotting of P-AKT, AKT, P-PI3K, PI3K, P-P38, P38, P-eNOS, eNOS, P-NF-κB, NF-κB, JNK, and ERK1/2 in vivo. **d** Statistical analysis demonstrated that the phosphorylation of the signaling pathway of PI3K/AKT/eNOS/P-38/NF-κB was highly upregulated in the N-ADSCs group as compared to the ADSCs group in vivo, but not JNK or ERK1/2; *P < 0.05. ADSC, adipose-derived stem cell; N-ADSC netrin-1-transfected ADSC

Western blotting demonstrated that the phosphorylation of the PI3K/AKT/eNOS/P-38/NF-κB signaling pathway was highly upregulated in the N-ADSCs group as compared to the ADSCs group both in vitro and in vivo (Fig. 9a, c). Statistical analysis demonstrated high upregulation of the signaling pathway in the N-ADSCs than the ADSCs group both in vitro and in vivo (Fig. 9b, d). On the other hand, the expression of ERK1/2 and JNK was not upregulated in the N-ADSCs as compared to the ADSCs group, putatively indicating that the MAPK signaling pathway might involve P-38-MAPK in N-ADSCs but neither ERK1/2-MAPK or JNK-MAPK. In addition, the activation of PI3K/AKT/eNOS is critical for the proliferation, differentiation, and anti-apoptosis of ADSCs [27]. Netrin-1 can restore cell injury and impaired angiogenesis in vascular endothelial cells under high glucose via the PI3K/AKT/eNOS signaling pathway [28]. Also, P38-MAPK was confirmed to be related to cell survival, differentiation, and migration [29]. Moreover, NF-κB was anti-apoptotic and upregulated the expression of VEGF, which is beneficial for angiogenesis. These findings demonstrated the significant function of Netrin-1 in regulating the survival, proliferation, migration, adhesion, and angiogenesis of ADSCs both in vitro and in vivo.

## Discussion

In the current study, ADSCs were obtained from mice adipose tissues; an efficient and stable system was established for producing N-ADSCs using gene transfection. Netrin-1 improved the proliferation, migration, adhesion, and angiogenesis of ADSCs and prevented the apoptosis induced by high glucose. Furthermore, N-ADSCs were implanted into type 2 diabetic mice with sciatic denervation. The N-ADSCs group demonstrated a significantly higher laser Doppler perfusion index than the ADSCs and the PBS control groups. The histological assay demonstrated that N-ADSC cells survived in the chronic ischemic muscles, and some differentiated into endothelial cells and formed small capillaries. The microvessel density of the hindlimb muscles was elevated in the N-ADSCs group as compared to the ADSCs and PBS control groups. The mechanisms underlying the improvement in survival, differentiation, migration, and revascularization of ADSCs might be associated with the upregulated PI3K/AKT/eNOS/P-38/NF-κB signaling pathway.

Several studies indicated adipose tissues as an abundant reserve of ADSCs; these stem cells harbor the potential of multiple differentiation, tissue restoration, and organ functions [30–32]. However, a large number of transplanted cells did not bring about a satisfactory treatment effect. And as shown in our study, ADSCs cultured and passaged to P6 demonstrated a pattern of serious aging and the reduction of stemness would result in a lower ability of proliferation, differentiation, and migration. So, we could only choose ADSCs of a narrow range of generations between P3 and P5 for the study. Moreover, DM or hyperglycemia limits the angiogenic effect of ADSCs, rendering the effective treatment of chronic DPNV as challenging. Thus, an improvement in the survival, differentiation, and migration ability of stem cells after transplantation is essential. Herein, we selected Netrin-1, a factor involved in both the functional activity of the nerves and the blood vessel system. In addition, several studies have shown its crucial role in the survival, adhesion, migration, proliferation, and differentiation of endothelial cells, nerve cells, and stem cells as well as inhibition of apoptosis. Ke et al. studied the positive effect of Netrin-1 on BMSCs in proliferation, migration, tube formation, and promotion in revascularization of rat limb ischemia. The study measured the plasma and tissue levels of VEGF and confirmed the upregulation of VEGF as the leading cause of the positive effect of Netrin-1-carrying BMSCs in the revascularization of rat limb ischemia [33].

The in vitro part of the current study revealed the upregulation of Netrin-1 on the viability, migration, adhesion, and differentiation of ADSCs and the underlying signaling pathway. The exogenous genes on the adenoviruses are not inserted into the cell genome, designating it as a superior and safer method than lentiviruses for clinical use [34]. Therefore, the *NTN-1* gene was transfected into ADSCs by adenoviruses. Western blotting assay also demonstrated the overexpression of Netrin-1 in transfected ADSCs and almost no expression in the control group. Netrin-1 enhanced the proliferation and prevented the apoptosis of ADSCs under high glucose. Western blotting showed that the ratio of Bcl-2/Bax was higher in N-ADSCs than in ADSCs. Previous studies revealed the function of Netrin-1 in inhibiting the apoptosis of mesenchymal stem cells (MSCs) via the DCC/AKT signaling pathway under hypoxia [35]. Netrin-1 was also shown to improve the adhesion, migration, and differentiation of ADSCs in the current results.

The in vivo immunofluorescence and histological assays proved that N-ADSCs survived more in the chronic ischemic muscles than ADSCs while some also differentiated into endothelial cells and formed small capillaries. This phenomenon increased the density of microvessels and the laser Doppler perfusion index, which together indicated an enhanced function of the hind limbs. Previous studies revealed that differentiation and paracrine effect are the two major mechanisms of ADSCs that have gained increasing attention recently [36]. The outcome of the current study proved that the overexpression of Netrin-1 in ADSCs greatly caused the overexpression of a range of growth factors and cytokines (for example, VEGF, HGF, bFGF, and PDGF), which, in turn, reduced the pathological remodeling of the hindlimb ischemia muscles and promoted the regeneration of the blood vessels. Nonetheless, highly expressed Netrin-1 is a robust angiogenesis regulator that restores cell injury and impaired angiogenesis in vascular endothelial cells under high glucose via PI3K/AKT-eNOS signaling pathway [28]. Moreover, we identified a mixed PI3K/AKT/eNOS/P-38/NF-κB signaling pathway that was found to be crucial for the proliferation, differentiation, anti-apoptosis, adhesion, and migration of ADSCs. But it was a preliminary exploration of the underlying mechanisms, and the specific mechanisms of the therapeutic effects of ADSCs in promoting angiogenesis are needed to be further clearly elucidated. Notwithstanding its limitations, the current study proved that Netrin-1 could improve the survival, migration, and treatment effect of ADSCs. Taken together, the outcomes provided a novel insight into the treatment of DPNV diseases.

## Conclusions

The present study showed that Netrin-1 is crucial for inhibiting the apoptosis of ADSCs and promoting the survival, adhesion, migration, and differentiation in vitro. After implantation into the chronic ischemic hindlimb of type 2 diabetic mice of sciatic denervation, N-ADSCs promoted the laser Doppler perfusion index and revascularization. The mechanisms of Netrin-1 on ADSCs were related to the activation of the PI3K/AKT/eNOS/P-38/NF-κB signaling pathway, as well as the secretion of cytokines and growth factors. Thus, Netrin-1 is referred to as a potential treatment factor for diabetic peripheral neurovascular diseases with respect to gene-based stem cell therapy.

## Abbreviations

ADSCs: Adipose-derived stem cells
b-FGF: Basic fibroblast growth factor
BMSCs: Bone marrow-derived stem cells
CCK-8: Cell Counting Kit-8
CLI: Critical limb ischemia
DM: Diabetes mellitus
DMEM: Dulbecco’s modified Eagle’s medium
DPNVs: Diabetic peripheral neurovascular diseases
EGF: Epidermal growth factor
EPCs: Endothelial progenitor cells
FBS: Fetal bovine serum
GFP: Green fluorescent protein
HEK293: Human embryonic kidney cell line 293
HGF: Hepatocyte growth factor
IGF-1: Insulin-like growth factor-1
MOI: Multiplicity of infection
N-ADSCs: Netrin-1-transfected ADSCs
*NTN-1*: Netrin-1 gene
OD: Optical density
PBS: Phosphate-buffered solution
PDGF: Platelet-derived growth factor
PET: Polyethylene terephthalate
PI: Propidium iodide
T2DM: Type 2 diabetes mellitus
TNF-α: Tumor necrosis factor-alpha
VEGF: Vascular endothelial growth factor
WT: Wild-type
